# Comparison of Conventional Chemotherapy, Stealth Liposomes and Temperature-Sensitive Liposomes in a Mathematical Model

**DOI:** 10.1371/journal.pone.0047453

**Published:** 2012-10-17

**Authors:** Astrid Gasselhuber, Matthew R. Dreher, Frank Rattay, Bradford J. Wood, Dieter Haemmerich

**Affiliations:** 1 Department of Pediatrics, Medical University of South Carolina, Charleston, South Carolina, United States of America; 2 Center for Interventional Oncology, Radiology and Imaging Sciences, Clinical Center, National Institutes of Health, Bethesda, Maryland, United States of America; 3 Institute for Analysis and Scientific Computing, Vienna University of Technology, Vienna, Austria; 4 Department of Bioengineering, Clemson University, Clemson, South Carolina, United States of America; University of Helsinki, Finland

## Abstract

Various liposomal drug carriers have been developed to overcome short plasma half-life and toxicity related side effects of chemotherapeutic agents. We developed a mathematical model to compare different liposome formulations of doxorubicin (DOX): conventional chemotherapy (*Free-DOX*), Stealth liposomes (*Stealth-DOX*), temperature sensitive liposomes (TSL) with intra-vascular triggered release (TSL-i), and TSL with extra-vascular triggered release (TSL-e). All formulations were administered as bolus at a dose of 9 mg/kg. For TSL, we assumed locally triggered release due to hyperthermia for 30 min. Drug concentrations were determined in systemic plasma, aggregate body tissue, cardiac tissue, tumor plasma, tumor interstitial space, and tumor cells. All compartments were assumed perfectly mixed, and represented by ordinary differential equations. Contribution of liposomal extravasation was negligible in the case of TSL-i, but was the major delivery mechanism for *Stealth-DOX* and for TSL-e. The dominant delivery mechanism for TSL-i was release within the tumor plasma compartment with subsequent tissue- and cell uptake of released DOX. Maximum intracellular tumor drug concentrations for *Free-DOX*, *Stealth-DOX*, TSL-i, and TSL-e were 3.4, 0.4, 100.6, and 15.9 µg/g, respectively. TSL-i and TSL-e allowed for high local tumor drug concentrations with reduced systemic exposure compared to *Free-DOX*. While *Stealth-DOX* resulted in high tumor tissue concentrations compared to *Free-DOX*, only a small fraction was bioavailable, resulting in little cellular uptake. Consistent with clinical data, *Stealth-DOX* resulted in similar tumor intracellular concentrations as *Free-DOX*, but with reduced systemic exposure. Optimal release time constants for maximum cellular uptake for *Stealth-DOX*, TSL-e, and TSL-i were 45 min, 11 min, and <3 s, respectively. Optimal release time constants were shorter for MDR cells, with ∼4 min for *Stealth-DOX* and for TSL-e. Tissue concentrations correlated well quantitatively with a prior in-vivo study. Mathematical models may thus allow optimization of drug delivery systems to achieve a better therapeutic index.

## Introduction

**Figure 1:**
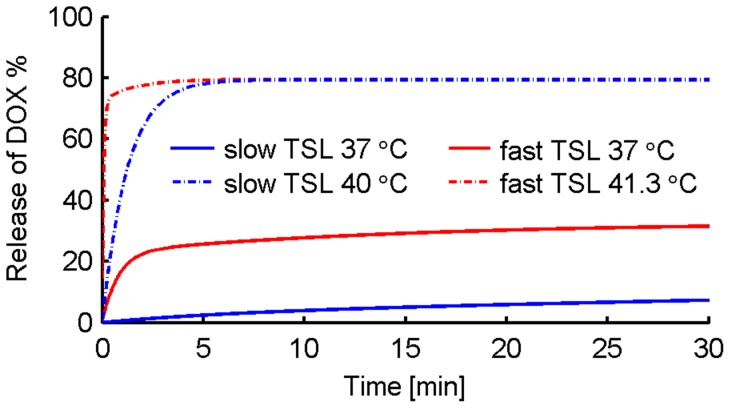
Release of DOX from different TSL at normal body temperature of 37°C and at ∼42°C. Release data adapted from prior studies [10], [13].

Current chemotherapy may be improved if sufficient levels of drug were obtained in the tumor while at the same time limiting system toxicity. Doxorubicin (DOX) is a clinically used chemotherapy agent with dose limiting toxicities [1] and a short plasma half life of five to ten minutes [2]. To overcome short plasma half-life of DOX and to reduce systemic toxicity, pegylated Stealth liposomal drug carriers for DOX (e.g., Doxil®) have been developed [3], allowing for long circulation times, up to several days [4], [5]. Stealth liposomes, such as Doxil, remain an excellent example of a drug delivery system with reduced toxicity, but there have been limited benefits in terms of clinical efficacy [6], [7], [8]. A different liposomal approach was first proposed in the late 1970s by Yatvin and colleagues [9] called temperature sensitive liposomes (TSL). TSL rapidly release their content upon heating (within seconds to minutes) [10], [11], [12], while at body temperature the drug is somewhat stably encapsulated (Figure 1). Therefore, TSL in combination with heating of the target region can selectively enhance bioavailability of the drug locally while minimizing systemic exposure.

In the last decade there has been increased interest in the TSL-based delivery, in part due to advances in image-guided hyperthermia applicators. The TSL approach requires the perfect marriage of liposomal properties, in terms of plasma pharmacokinetics and temperature dependent release, with a hyperthermia applicator that generates accurate and homogeneous spatial temperature distributions. TSL have been successfully combined in both preclinical and clinical studies with heat-based thermal therapies including radiofrequency ablation [13], [14], ultrasound hyperthermia [15], [16], [17], and microwave hyperthermia [18]. Although many reports suggest potential of a TSL based approach, the optimal combination of TSL and hyperthermia applicator properties for a given tumor type remains unknown. For example, TSL were formulated to provide ultrafast release within seconds [13], [19] while other approaches use a longer circulating liposome with longer release times within minutes to hours [20], [21], [22]. Ultrafast release TSL may facilitate an intravascular triggered tumor delivery paradigm, but more stable long circulating liposomes may first accumulate in the tumor region prior to substantial temperature activated drug release.

One difficulty in uncovering an optimal combination of TSL and hyperthermia applicators is that drug delivery is determined by the interplay of several transport mechanisms affected by a large number of parameters, e.g. vascular density, permeability, perfusion, and rate of cellular uptake to name a few. While it is not possible to systematically examine (or in many cases even measure) the influence of these parameters with in vivo studies, computational models offer the unique ability to efficiently perform such a multi-parameter analysis. In prior studies, mathematical models have described the pharmacokinetics of DOX resulting from different drug delivery methods [23], [24], [25], including intravascular and extravascular triggered release from TSL [13], [17], [26]. Mathematical analysis of drug delivery kinetics can thus identify the key parameters that affect drug distribution. Furthermore, these models may facilitate optimization of parameters, such as drug release rate and plasma half-life. The objective of this study was to mathematically model and compare standard chemotherapy to Stealth liposomes and TSL with different release time constants triggered intra- or extra-vascularly, and to determine plasma- and tumor concentrations of bioavailable drug. Further we examined importance of liposome extravasation and changes in cellular uptake rate. These findings are significant in that they provide a basic foundation for current activate-able drug delivery approaches and essential guidance for future drug delivery system development.

## Materials and Methods

A mathematical model to simulate and compare drug delivery after administration of either liposomal encapsulated DOX or DOX alone was developed. Specifically, the following cases were modeled, where all drug types were administered as bolus injection at a dose of 9 mg/kg in mice (small animals were modeled, since for most drug formulations below only transport parameters from small animal studies data is available):

1. ***Free-DOX***: Doxorubicin alone (i.e. administered unencapsulated)
2. ***Stealth-DOX*** **(passive release):** Stealth liposomes at varying release time constants:
  - Release time constant *τ* = 454.4 h (corresponds to release time constant of Doxil)
  - Same release time constant as (a), but with assumption that no liposomal extravasation takes place to examine relative contribution of liposomal extravasation
  - Optimization of release time constant in order to maximize intracellular DOX tumor concentration

The release time constant describes the time after which ∼63% of content is released assuming exponential release time course.

1. ***TSL-e-DOX*** **: Extravascular triggered TSL**: Thermally sensitive Stealth liposome formulation with extravascular activated release: Liposomes are allowed to accumulate in tissue for 24 h, assuming long plasma half life (i.e. that of *Stealth-DOX*). Upon trigger, release at varying release time constants between 0 and 7 h were considered within plasma and interstitium to find optimal rate. Tumor was assumed to be uniformly heated to 42°C for 30 min to trigger release.
2. ***TSL-i-DOX*** **: Intravascular triggered TSL:** Release triggered by hyperthermia within plasma (i.e. triggered before liposomes can extravasate from vasculature into interstitium) immediately following administration. Two different formulations were modeled (see Figure 1):
  - fast-release TSL-i: release rate 0.3/s (i.e. complete release within ∼3–4 s) at 41.3°C [13]
  - slow-release TSL-i: release rate 0.025/s (i.e. complete release within a few min) at 42°C [10]

The release rates were calculated as linear approximation of the data in Figure 1 at t = 0 (initial slope). The assumption of linear release time course is adequate since *TSL-i* only release during the few seconds of tumor transit, where curves in Figure 1 are approximately linear. The tumor was assumed to be uniformly heated to 42°C for 30 min to trigger release during this period. For the release of DOX due to liposomal instability at 37°C a biexponential fit to experimental data was used (see release of DOX at 37°C shown in Figure 1).

1. ***Validation of Free-DOX, Stealth-DOX, TSL-i-DOX models***: A prior in-vivo study measured tissue DOX concentration 1 h after administration of different DOX formulations, with and without hyperthermia (1 h@42 °C) [12]. Kong et al studied two TSL formulations: the slow-release formulation from above (complete release within ∼2 min, release rate 0.025/s), and one with complete release after ∼1.5 h (release rate 0.00096/s). We implemented these release parameters in our model, and used same dose (5 mg/kg). We modeled *Free-DOX* and *Stealth-DOX* without hyperthermia, and the two TSL-formulations with hyperthermia. Tissue concentration after 1 h was compared between our model and this study by Kong et al [12].

In the following text we will always refer to the fast-release *TSL-i* formulation, except if specified otherwise.

**Figure 2:**
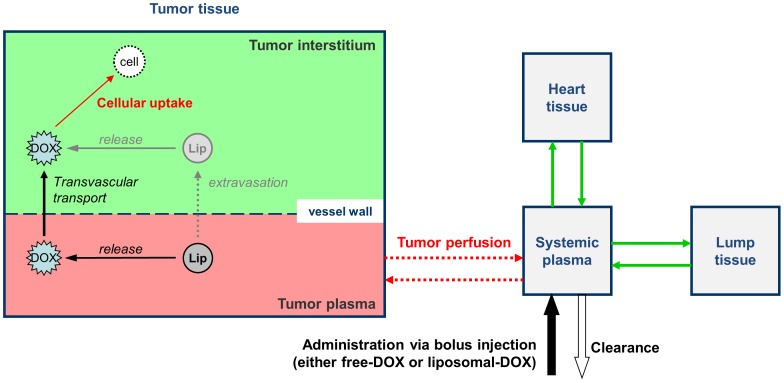
Pharmacokinetic multi-compartment model of DOX and liposome transport. Systemic plasma, heart, and other bodily tissues are modeled as single compartments. Tumor is modeled in more detail including plasma, interstitium (EES), and cells as separate compartments. Transvascular transport of both bioavailable DOX and liposomes is driven by the concentration difference between the tumor plasma and the EES of DOX/liposomes, as well as on the vascular permeability for DOX/liposomes, and vessel on surface area.

All simulations used the same multi compartment model, which included following compartments (see Figure 2):

- Systemic plasma compartment
- Aggregate body tissue compartment
- Heart tissue compartment (cardiac toxicity limits DOX lifetime exposure)
- Tumor compartment, which was further divided into:

o Tumor plasma compartment

o Tumor extravascular extracellular compartment (EES)

o Tumor intracellular compartment.

Compartments were assumed perfectly mixed, and for each compartment the DOX concentration was calculated over a 50 h period. Additionally, the liposomal (i.e. encapsulated) DOX concentration was calculated for the plasma and tumor EES. As presented in Figure 2, liposomal release of drug, extravasation of liposomes from tumor vasculature into the tumor EES, transvascular transport of free drug and cancer cell uptake were modeled similar to prior studies [17], [23], [27], [28].

The aggregate body tissue compartment and systemic plasma compartment were assumed at 37°C body temperature, and a uniformly distributed blood perfusion w_0_ = 108 ml/100 ml/min was assumed.

All simulations were done with Matlab (R2009a) Software. The model was implemented via a system of ordinary differential equations (ODEs), similar to a prior publication [13]. The complete set of equations is listed and described in detail in the supplementary data.

Below, the assumptions underlying the model, and descriptions on how different processes were modeled, are presented:

- *TSL-DOX (TSL-i, TSL-e)*: Liposomal DOX concentration within the systemic plasma considered release of DOX at body temperature (due to liposomal instability), body clearance of both DOX and liposomes, and triggered release within the tumor (either intra-, or extravascularly for *TSL-i* and *TSL-e*, respectively). We assumed uniform transport parameters within the tumor (i.e. no spatial heterogeneity was considered).
- *Stealth-DOX*: This formulation releases DOX in whole body plasma rather than just in the tumor plasma and interstitium (EES) as for *TSL-DOX*. A constant release time constant of liposomal release within plasma as well as tumor interstitium was assumed.
- Tumor sub-compartments: Tumor tissue was modeled in more detail than other tissues, where plasma, interstitial, and intracellular regions were each represented separately (Figure 2). The transport of liposomes as well as unencapsulated DOX were modeled as diffusion based processes, and thus depend on the concentration difference between plasma and EES, as well as on apparent vascular permeability (*P*) and vascular surface area (*S*):

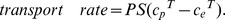



Even though transport processes other than diffusion potentially contribute (e.g. convection), this approach is appropriate as long as overall transport can be represented accurately by an apparent permeability, and this method has been employed in numerous prior studies [17], [23], [29].

While uptake of liposomes by macrophages and subsequent release of DOX is another possible mechanism enhancing tissue concentration [30], [31], this mechanism is not considered here. For *TSL-i*, macrophage uptake is not expected to have significant impact due to the comparably slow rate, though it may contribute in the case of *Stealth-DOX* and *TSL-e*.

- Although it is known that DOX binds extensively (∼70%) to plasma proteins [2], experimental data [32] suggest that plasma protein binding of DOX is weak and does not affect transvascular transport significantly. Thus we did not include any protein binding of DOX in this current modeling study.
- Note that vascular permeability for liposomes is by three orders of magnitude lower than permeability for unencapsulated DOX (Table 1). Further, rate of tumor perfusion – which delivers liposomes and unencapsulated DOX to tumor as well as removes those – affects liposomal and drug transport and is considered.
- DOX cell uptake was modeled according to a mathematical cell uptake model suggested in a prior publication [27], which is based on in-vitro studies in human non-small cell lung tumor cells [33]. For modeling MDR resistant cells, DOX cellular efflux was increased by a factor of ten compared to non-resistant cells [24].
- Maximum tumor intracellular concentration was considered as measure of efficacy of a particular liposomal formulation, since intracellular concentration has been shown to directly correlate with cell survival independent on cell type, contrary to EES concentration [34].
- The total tumor tissue drug concentration was calculated by weighted averaging of concentrations in EES (unencapsulated and liposomal) and tumor cells, considering the volume fraction of each compartment. The tumor concentration of bioavailable DOX was calculated by weighted averaging (based on EES and intracellular volume fractions) of concentrations of unencapsulated DOX in tumor EES, and tumor cells.
- Body and heart tissue compartments: The systemic plasma compartment concentration considered inflow and outflow from tumor, as well as clearance, and uptake by body tissue. All body tissues except tumor and heart were lumped together in one compartment (Figure 2), and transport to and from tissue were described each by a rate constant. No specific tissue types, and no separation into EES and cellular compartments were considered. Heart tissue was modeled in the same manner, but with different transport rate constants than the lump tissue. Heart tissue concentration served as marker of cardiac toxicity for our purposes, which limits DOX lifetime exposure.
- For all liposomal carriers, we assumed a bolus injection at time = 0, and perfect mixing within systemic plasma after administration. Similarly, for administration of *Free-DOX*, a bolus injection was assumed, with initial volume of distribution defining initial DOX concentration.

**Table 1:** Primary model parameters.

Symbol	Description	Value	Source
*BW*	Body weight for mice	20 g	N/A
*VD*	Volume of distribution for free DOX	19e-6 m^3^	Calculated with data from [35]
*D*	Total dose of encapsulated DOX injected	0.18 mg	9 mg/kg * bodyweight (assumed )
*PS_DOX_*	Permeability surface area product for DOX	4.9e-3 s^−1^	[25]
*V_b_^B^*	Total blood volume in body	1.69 mL	*V_b_^B^* = 84.7 mL/kg *BW* [36]
*V_p_^B^*	Volume of systemic plasma	1.12 mL	*V_p_^B^* = 56.1 mL/kg *BW* [36]
*v_v_^T^*	Volume fraction of tumor vascular space	0.092	[37]
*v_p_^T^*	Volume fraction of tumor plasma space	0.0745	*v_v_^T^*(1-*Hct_tumor_*)
*v_e_^T^*	Volume fraction of tumor EES	0.454	[38]
*v_i_^T^*	Volume fraction of tumor intracellular space	0.454	(1-*v_v_^T^*-*v_e_^T^*)
*R*	Release rate of DOX from TSL-i during heat,for fast TSL-i if *F_pv_^T^*>*R*for slow TSL-i if *F_pv_^T^*>*R*if *F_pv_^T^*< = *R*	0.3 s^−1^0.025 s^−1^F_pv_^T^	calculated
*R_37_*	Release rate of DOX from TSL-i at 37°C	variable [s^−1^]	see Fig. 1
*w_0_*	Blood perfusion	108 ml/100 ml/min	[39]
*P_L_S*	Permeability surface area product for liposomes	4e-6 s^−1^	[40], [41], [42]
*V^T^*	Volume of tumor (diameter of 2 mm)	4.189e-3 mL	
*V_p_^T^*	Volume of tumor plasma	3.12e-4 mL	V^T^*v_p_^T^
*τ*	Time constant for DOX release from Doxil (equal for plasma and EES)	454.4 h	[4], [5]
*k_e_Lip_*	clearance Stealth liposomes	0.0339 h^−1^	[5]

Table 1 shows the primary model parameters, including mouse physiological variables. A complete list of parameters is presented in the supplementary data.

## Results

**Figure 3:**
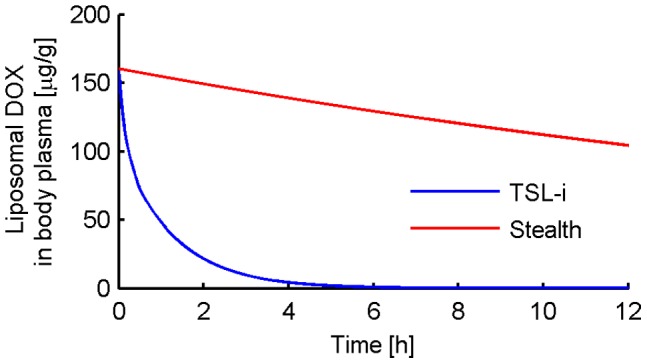
Liposomal DOX plasma concentration for Stealth liposomes and *TSL-i* over a time period of 12 h. Shorter *TSL-i* half-life results from reduced stability at body temperature compared to Stealth liposomes.

**Figure 4:**
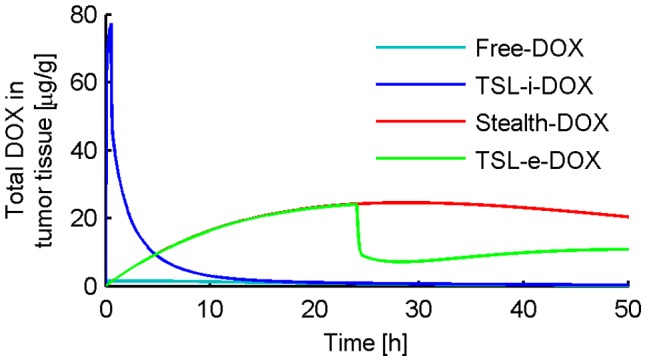
Total DOX (including liposomal and bioavailable) in tumor tissue (EES and intracellular) for different DOX formulations.

**Figure 5:**
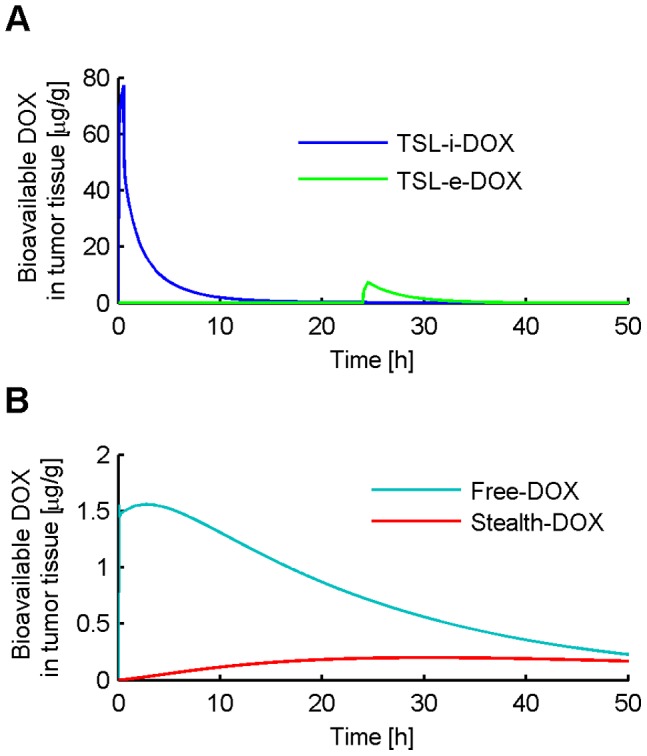
Bioavailable tumor tissue DOX concentration for (A) *TSL-i-DOX* and *TSL-e-DOX*, and (B) *Free-DOX* and *Stealth-DOX* over time.

**Figure 6:**
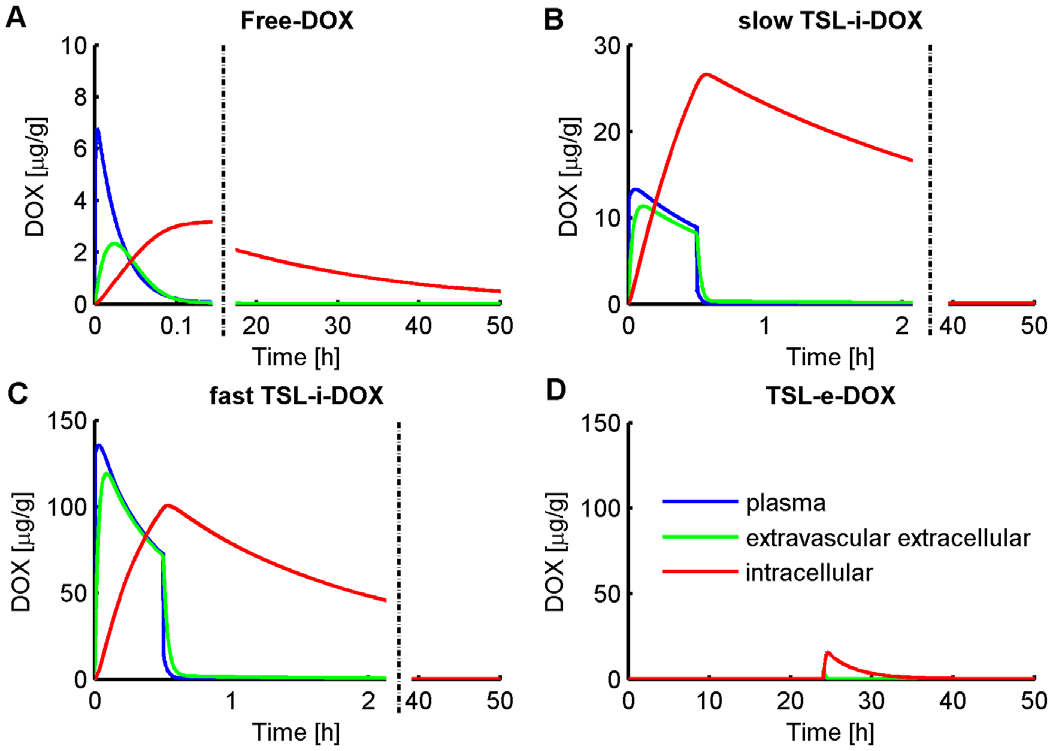
Plasma, EES- and intracellular tumor concentration time courses of bioavailable DOX for (A) *Free-DOX*, (B) slow-release *TSL-i-DOX*, (C) fast-release *TSL-i-DOX*, and (D) *TSL-e-DOX*. For (D), 24 h extravasation was considered with subsequent triggered release from TSL-e in interstitium and plasma. Note that EES and intracellular concentrations are relative to EES and cell volume, i.e. would need to be multiplied by appropriate volume fractions for conversion to tissue concentration.

**Figure 7:**
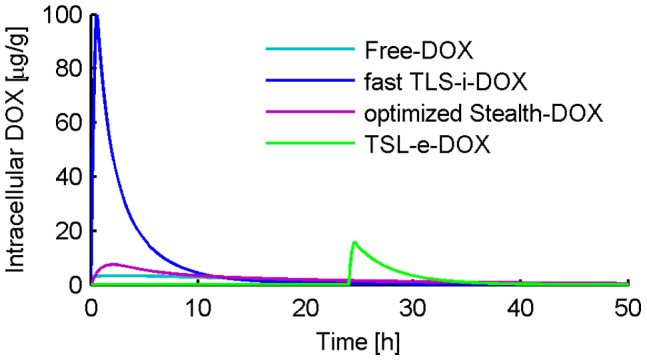
Comparison of intracellular tumor DOX concentration for different DOX formulations.

Figure 3 shows the concentration of liposomal DOX in body plasma resulting from administration of *Stealth-DOX* and *TSL-i-DOX*, showing comparably shorter plasma half-life of *TSL-i* due to lower stability of this fast-release TSL formulation (see Figure 1). The highest peak tumor concentration of total DOX (i.e. liposomal and released DOX) was reached with *TSL-i-DOX*, followed by *Stealth-DOX* and *TSL-e-DOX* (Figure 4 and Table 2). For *Stealth-DOX*, only a small fraction of total DOX in tumor was bioavailable (Figure 5B); bioavailable tumor DOX concentration was highest for *TSL-i* with a peak value of 77.34 µg/g (Figure 5A and Table 2). Figure 6 compares time course of bioavailable DOX concentration in plasma, EES, and inside tumor cells, each for *Free-DOX*, *TSL-i*, and *TSL-e*. Plasma and EES concentrations of bioavailable DOX equilibrate within ∼2 min due to comparably high vascular permeability of unencapsulated DOX. *Free-DOX* results in short drug exposure of cells, and low cellular uptake (Figure 6A). *TSL-i* allow for extended duration of high plasma concentration within the tumor, with considerably higher cell uptake. For *TSL-i*, rapid release formulations (within seconds) are ideal (Figure 6C), allowing for higher EES concentration and cell uptake than those with slower release (Figure 6B). *TSL-e* did not achieve as high cellular uptake as *TSL-i,* as most drug released within the interstitium by extravasated *TSL-e* diffused back into the vasculature, and was carried away by blood flow. Figure 7 compares time course of intracellular concentration of all formulations. The maximum intracellular tumor drug concentration was highest for *TSL-i* (100.55 µg/g) compared to *TSL-e* (15.9 µg/g), *Free-DOX* (3.39 µg/g) and *Stealth-DOX* (0.44 µg/g) (Figure 7 and Table 2). Stealth liposomes released their content slowly; therefore the maximum bioavailable DOX concentration was reached much later than with other formulations (Table 2).

**Table 2:** Comparison between different DOX formulations for both DOX sensitive cells and MDR cells.

DOX sensitive cells	*Free-DOX*	*TSL-i-DOX*(fast)	*TSL-i-DOX*(slow)	*Stealth-DOX*(Doxil)	*Stealth-DOX*(optimized for DOX sensitive cells)	*TSL-e-DOX*(optimized for DOX sensitive cells)
Peak bioavailable tumor tissueconcentration of DOX	1.6 µg/gat t = 2.8 h	77.3 µg/gat t = 30 min	15.2 µg/gat t = 30 min	0.2 µg/gat t = 30.3 h	3.5 µg/gat t = 2 h	7.5 µg/gat t = 24.5 h
Peak cardiac tissue concentrationof DOX	6.6 µg/gat t = 8 min	1.8 µg/gat t = 32 min	0.7 µg/gat t = 1.8 h	0.1 µg/gat t = 22.9 h	4.9 µg/gat t = 1.8 h	0.2 µg/gat t = 24.6 h
Peak bioavailable tumor tissueconcentration/Peak cardiactissue concentration	0.2	42.1	22.1	2.4	0.7	47.9
Peak intracellular tumor tissueconcentration of DOX	3.4 µg/gat t = 2.8 h	100.6 µg/gat t = 32 min	26.6 µg/gat t = 34.3 min	0.4 µg/gat t = 30.3 h	7.5 µg/gat t = 2 h	15.9 µg/gat t = 24.5 h
Peak systemic plasma concentrationof free DOX	9.5 µg/gat t = 0	0.4 µg/gat t = 2.8 min	0.1 µg/gat t = 3.2 min	0.001 µg/gat t = 20.2 h	0.3 µg/gat t = 5.3 min	0.01 µg/gat t = 24.1 h
Plasma AUC of unencapsulated DOX	1.2 µg h/g	0.3 µg h/g	0.2 µg h/g	0.1 µg h/g	1.1 µg h/g	0.1 µg h/g
AUC of bioavailable tumorconcentration of DOX	40 µg h/g	157.9 µg h/g	53.8 µg h/g	7.8 µg h/g	50.6 µg h/g	30.8 µg h/g
AUC of bioavailable tumorconcentration of DOX/Peak cardiactissue concentration	6.0	85.9	78.1	92.3	10.4	196.2
**MDR cells**						
Peak bioavailable tumor tissueconcentration of DOX	1.5 µg/gat t = 2 min	63.7 µg/gat t = 7 min	8.2 µg/gat t = 14 min	0.02 µg/gat t = 26.4 h	1.0 µg/gat t = 32 min	4.0 µg/gat t = 24.1 h
Peak cardiac tissue concentrationof DOX	6.6 µg/gat t = 8 min	1.8 µg/gat t = 32 min	0.7 µg/gat t = 1.8 h	0.1 µg/gat t = 22.9 h	4.9 µg/gat t = 1.8 h	0.2 µg/gat t = 24.6 h
Peak bioavailable tumor tissueconcentration/Peak cardiactissue concentration	0.2	34.6	11.9	0.3	0.2	23.6
Peak intracellular tumor tissueconcentration of DOX	2.1 µg/gat t = 6 min	26.8 µg/gat t = 16 min	6.7 µg/gat t = 22 min	0.04 µg/gat t = 26.4 h	1.8 µg/gat t = 34 min	4.5 µg/gat t = 24.2 h
Peak systemic plasma concentrationof free DOX	9.47 µg/gat t = 0	0.35 µg/gat t = 3 min	0.08 µg/gat t = 3 min	0.0012 µg/gat t = 20.1 h	0.28 µg/gat t = 5 min	0.01 µg/gat t = 24.1 h
Plasma AUC of unencapsulated DOX	1.2 µg h/g	0.3 µg h/g	0.2 µg h/g	0.05 µg h/g	1.1 µg h/g	0.06 µg h/g
AUC of bioavailable tumorconcentration of DOX	4.4 µg h/g	31.8 µg h/g	5.8 µg h/g	0.9 µg h/g	5.4 µg h/g	2.6 µg h/g
AUC of bioavailable tumorconcentration of DOX/Peak cardiactissue concentration	0.7	17.3	8.3	10.9	1.1	15.1

Note that plasma, EES and intracellular concentrations are relative to volume fraction (i.e. plasma, EES, or cellular fraction). EES and intracellular concentrations would have to be multiplied by appropriate volume fractions for conversion to tissue concentration.

The highest cardiac tissue concentration resulted from the administration of *Free-DOX*. Peak cardiac tissue concentration of DOX in the case of *TSL-i* (1.84 µg/g) was only about a third of the peak concentration resulting from administration of *Free-DOX* (6.62 µg/g) (Table 2), and was mainly due to leakage at body temperature (Figure 1). The best ratio between peak bioavailable tumor tissue drug concentration and cardiac tissue concentration was reached with *TSL-e* (Table 2).

**Figure 8:**
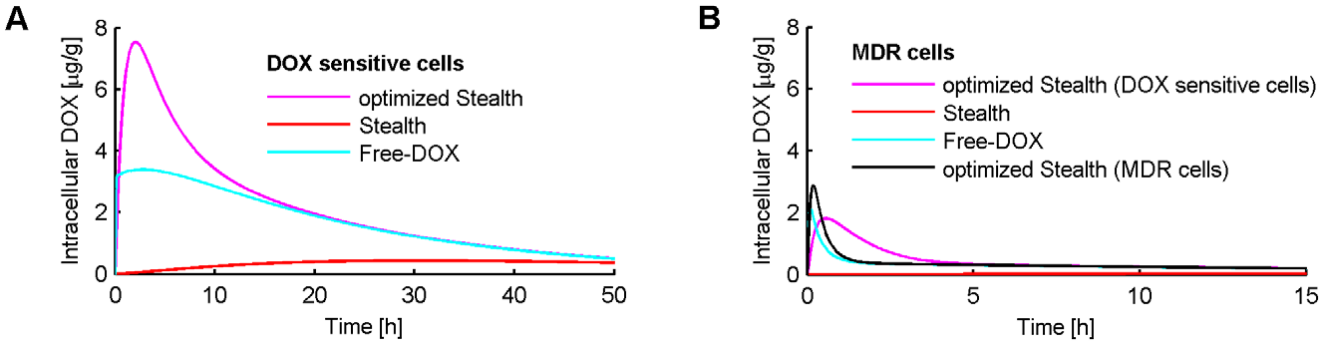
(A) Comparison of *Free-DOX*, Doxil (*τ* = 454.4 h), and optimized Stealth liposomes (*τ* = 45 min) in DOX-sensitive cells, showing intracellular concentration time course. (B) Considerably lower intracellular concentrations are achieved with same formulations in MDR cells. Further, the optimal liposomal release time constant is considerably lower (*τ* = 4 min) for MDR cells.

**Figure 9:**
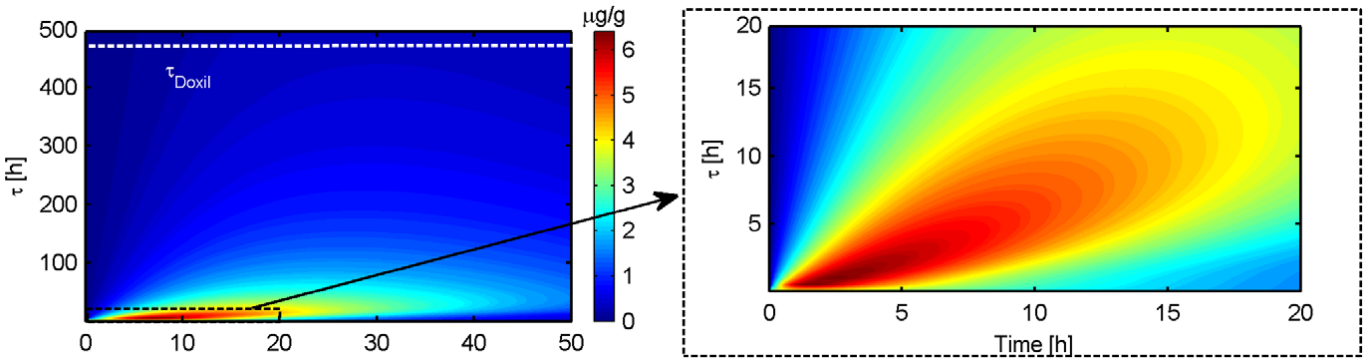
Optimization of release time constant (*τ*) for Stealth liposomes: Max intracellular tumor drug concentration was obtained for a release time constant of 45 min. Clinical Stealth DOX formulation Doxil (*τ* = 454.5 h) is shown for comparison as white dashed line.

**Figure 10:**
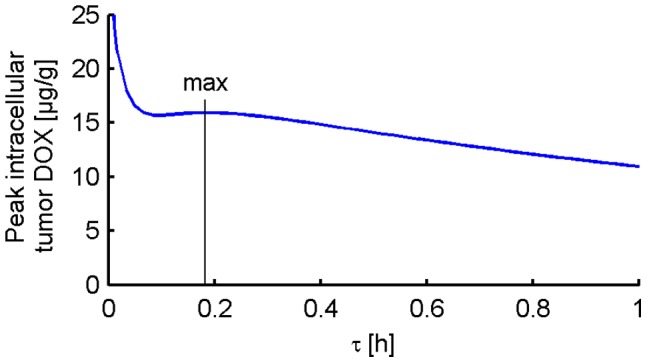
Optimization of release time constant for TSL-e. The optimum (optimum indicated by line at ∼11 min) is where contribution of release from extravasated TSL-e towards drug delivery is maximal (intended delivery mechanism). While higher intracellular concentrations are observed at very short release time constants, this is due to contribution from intravascular release, i.e. the *TSL-e* become equivalent to *TSL-i*.

The optimization of Stealth liposomes in order to maximize the intracellular tumor drug concentration resulted in an optimal release time constant of *τ* = 45 min compared to clinically used *Stealth-DOX* (Doxil) which has a release time constant of *τ* = 454.5 h [4] (Figure 8, Figure 9). The optimized Stealth liposomes reached a peak intracellular drug concentration of 7.54 µg/g, compared to 0.44 µg/g with Doxil. However, the maximum plasma concentration and AUC were also higher in the case of optimized Stealth liposomes compared to Doxil (see Table 2) suggesting higher toxicity. The ratio between maximum DOX concentration in tumor tissue to cardiac tissue was 0.71 and 2.35 for optimized Stealth and Doxil, respectively (Table 2). Figure 8 and Figure 9 show tumor intracellular DOX concentration for different *Stealth-DOX* release time constants (*τ* = 1–500 h), with optimum at τ = 45 min. The release time constant was also optimized for *TSL-e* (Figure 10), and was optimal at *τ* = 10.9 min. Note however that this optimal time constant is tied to the 30-min hyperthermia duration. For extended hyperthermia durations longer than 4 h, the optimum release time constant approaches ∼30 min.

All simulations were repeated for the case of MDR tumor cells, which resulted, as expected, in lower intracellular drug concentrations in all cases, but showed similar levels of EES and plasma DOX concentrations (results shown in Figure 8 and Table 2) as for DOX-sensitive cells. In the case of MDR cells, the optimal release time constant for Stealth liposomes and *TSL-e* was considerably shorter (*τ* = 4 min in both cases, shown in Figure 8) in order to maximize intracellular drug concentration.

When we modified the model in that we didn’t allow extravasation of liposomal drug into the tumor EES demonstrated negligible contribution of TSL extravasation in the case of *TSL-i*. In contrast, for *Stealth-DOX*, tumor intracellular concentration was reduced 3.7 fold if extravasation was not allowed. Table 2 summarizes concentrations for all formulations.

## Discussion

Despite dose limiting toxicities, DOX is one of the most commonly used anticancer drugs. Several recent studies show that liposomal encapsulated DOX can overcome some of these negative side effects of unencapsulated DOX, resulting in reduced toxicity but not necessarily higher treatment efficacy [43]. Stealth liposomes release their content slowly over time and have prolonged circulation times, up to several days. In contrast, TSL are designed to release most of their contents within seconds to minutes upon heating, whereas at normal body temperature only a small amount of drug is released (Figure 1). Thus the toxicity compared to *Free-DOX* can be reduced while increasing drug accumulation at the target site through locally triggered release.

In this study we used mathematical models to compare administration of conventional chemotherapy (unencapsulated DOX), DOX loaded TSL and Stealth liposomes in mice. Drug concentrations in plasma, normal tissue and tumor tissue, as well as various transport mechanisms were investigated. For all liposomal formulations, different release time constants were examined in order to maximize intracellular peak concentration of DOX. Intracellular DOX concentration was used as measure of efficacy, since it directly correlates with cell kill, contrary to EES concentration [33]. We examined the ratio of drug exposure between tumor tissue and cardiac tissue for each DOX formulation as a measure of selectivity.

### Free-DOX (Conventional Chemotherapy)

Unencapsulated DOX has a short initial plasma half life (∼1 min in mice, see Figure 6A) [44]. The administration of unencapsulated DOX results in a high initial peak plasma concentration followed by a fast decrease. Most of the drug is cleared out of the body before the tumor cellular compartment has sufficient time to take up substantial amounts of drug. Hence, peak EES concentration in the tumor compartment is 2.33 µg/g after 1.4 min, followed by a fast decrease according to the decrease of the plasma concentration (Figure 6A). Drug uptake by EES results from transvascular transport of drug, and is therefore limited by the short period of high plasma concentration. In fact, the time constant for DOX transvascular transport (i.e. equilibration between EES and plasma concentration) is about two minutes (Figure 6), which is significantly longer than the plasma half-life. That is for example why slow infusion of DOX is more effective than rapid bolus [23]. As tumor intracellular uptake of DOX occurs at a slower rate than the rate of transvascular transport, peak intracellular concentration of 3.39 µg/g occurs delayed after 2.8 h (Figure 7). Intracellular tumor drug concentration is low and heart tissue concentration high compared to liposomal formulations, as shown in Table 2.

### Stealth-DOX

**Figure 11:**
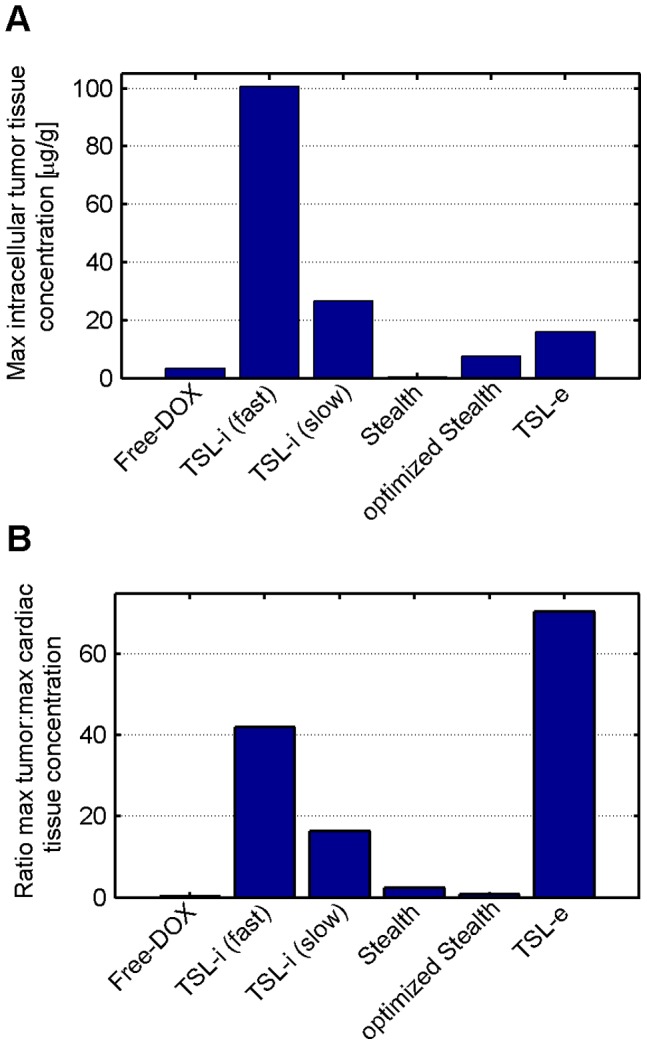
(A) Peak intracellular tumor tissue concentration of bioavailable DOX, and (B) ratio of peak bioavailable tumor tissue concentration to peak cardiac tissue concentration, presented for all DOX formulations.

Increasing the period for which EES DOX concentration is elevated would be expected to result in higher intracellular DOX concentrations. This is more likely to be obtained with liposomal DOX than with the administration of unencapsulated DOX. Liposomes extravasate from tumor vasculature and accumulate in tumor tissue due to their prolonged plasma circulation time and the higher permeability of tumor vasculature compared to most noncancerous tissues [45]. Our results show that for Stealth liposomes, extravasation is a dominant transport mechanism as indicated by a 3.7-fold reduction in intracellular DOX when liposome extravasation was not included in the model. Due to the slow release (*τ* = 454.4 h) of DOX from Stealth liposomes, the peak concentrations of DOX in the plasma and EES are low compared to concentrations after administration of *Free-DOX*. Several prior animal studies have shown increased tumor drug accumulation with Stealth liposomes compared to *Free-DOX* administration [46], [47], similar to our results (Figure 4). While more drug is accumulated in tumor tissue when administered via Stealth liposomes, importantly, most of the accumulated drug is present as encapsulated liposomal drug in the EES with only a small fraction released and being bioavailable (Figure 5B). This may explain why in general Stealth liposomes result in reduced toxicity, but not higher efficacy compared to free drug administration [43]. The reduced toxicity of Stealth liposomes compared to free drug was also observed here in terms of reduced cardiac tissue concentration (Table 2). El-Kareh and Secomb [23] compared different infusion times of DOX, as well as different release time constants of DOX from Stealth liposomes for 4 different doses. We performed a similar parametrical analysis for different release times *τ* of Stealth liposomes (Figure 9). For very small release time constants *τ* (<1 min) the results closely match those after the administration of *Free-DOX*. Similar to the prior study by El-Kareh and Secomb [23], our results show that the optimal release time constant of Stealth liposomes in order to maximize intracellular tumor concentration is 45 min, which is much lower than the release time constant of current clinically used Stealth liposomes (e.g. Doxil). However, these optimized Stealth liposomes also had higher plasma drug concentrations (peak concentration as well as AUC) compared conventional *Stealth-DOX*, which might implicate higher toxicity. Therefore not only intracellular tumor concentration, but also systemic toxicity should be considered in choice of the optimal release properties to achieve the optimal therapeutic index. Table 2 and Figure 11(B) show that the ratio between peak concentrations of tumor tissue and cardiac tissue is better for Doxil, than for the optimized Stealth liposomes. The optimal liposomal release rate also depends on the intracellular DOX uptake mechanism of cells, as this affects uptake rate. The cellular uptake mechanisms are likely to be different for different cell types (e.g. MDR tumor cells as discussed below), and therefore the presented results are only valid for the cell type we assumed in this study (human non-small cell lung tumor cells).

### TSL-i-DOX (Intravascular Triggered TSL)

The dominant mechanism of delivery by fast-releasing *TSL-i* is dissimilar from Stealth liposomes, in that DOX is released inside the tumor plasma compartment at the location of heating, and subsequently transported into the tissue’s EES as free drug. The extravasation of *TSL-i* into the EES is not relevant due to the short plasma half-life of *TSL-i* and slow extravasation rate of nanoparticles. The release of DOX from the fast-release TSL within systemic plasma at body temperature results in a higher systemic plasma drug concentration compared to Stealth liposomes, though still considerably lower than for administration of *Free-DOX* (Figure 6). Liposomal body plasma concentration of *TSL-i* decreases much faster compared to Stealth liposomes due to the lower stability of the fast-release *TSL-i* formulation (Figure 3). The mechanism for the shorter plasma half-life is unknown but it is most likely a combination of DOX leakage from the *TSL-i* and clearance of the *TSL-i* containing DOX. Tumor plasma concentration of bioavailable DOX is highest for fast-release *TSL-i* due to quick release of drug from heated TSL while the liposomes are transiting the heated tumor vasculature. Rapid intravascular release of DOX upon heating provides locally maintained elevated tumor intravascular concentration during heating (Figure 6C); this is conceptually similar to local infusion of unencapsulated drug into the tumor’s vascular supply. Thus, the systemic plasma serves as large reservoir of non-bioavailable drug that becomes bioavailable once entering the heated tumor.

**Figure 12:**
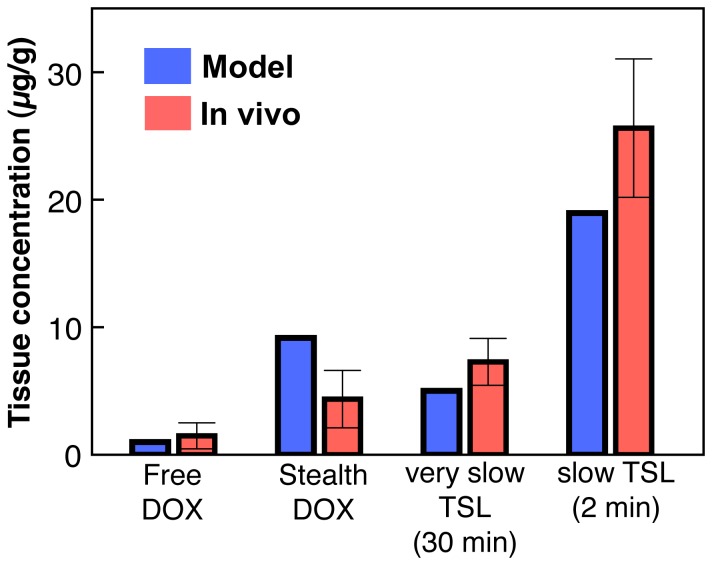
Comparison of mathematical model to a prior in-vivo study [12] (note that the fast *TSL-i* formulation discussed above was not employed in this prior study). *Free-DOX* and *Stealth-DOX* were compared without hyperthermia; for the two *TSL-i-DOX* formulations, 1 h hyperthermia was assumed. Administered dose was 5 mg/kg (compared to 9 mg/kg in all other models).

The amount of released drug within the heated region not taken up by the tumor, and carried by perfusion into the systemic plasma, is negligibly small compared to drug leaked from *TSL-i* systemically at body temperature; this is due to the much larger volume of the systemic plasma compared to tumor plasma compartment [48]. However, this intravascular release approach may be improved by using drugs that are completely extracted by the heated tissue. In addition to fast-release *TSL-i*, a slow-release *TSL-i* formulation [22] with release rate of *R* = 0.025 s^−1^ (complete release within a few minutes) was modeled, which resulted in a 2.7 fold lower heart tissue DOX concentration (due to lower leakage at body temperature) and 5.1 fold lower tumor tissue DOX concentration compared to the fast release *TSL-i* formulation. This is in agreement with a prior study, where very slow-release *TSL-i* (release within ∼1 h) showed no benefit over *Stealth-DOX*, compared to *TSL-i* that release within 1 min [12] (Figure 12). An ideal *TSL-i* would therefore have no release at body temperature, and very quick complete release (within ∼ a second) when heated.

### TSL-e-DOX (Extravascular Triggered TSL)

This TSL formulation combines the stability and long circulation time of Stealth liposomes with heat-activated release after liposomal tumor accumulation. Liposomes were allowed to extravasate for 24 h until they reach maximum EES concentration. At this time point we initiatde release of drug from the liposomes within the tumor EES and plasma by local heating (Figure 6D). Following release, some bioavailable DOX is taken up by cells, but most DOX diffuses back into plasma due to fast transvascular transport. Cellular DOX uptake is therefore lower than for *TSL-i*. The optimal release time constant for *TSL-e* was 11 min (Figure 10). While Figure 10 shows highest tumor drug uptake at very short release time constants, this is due to contribution of intravascular release (i.e. would correspond to *TSL-i* described below). The maximum indicated in Figure 10 is at the release rate where contribution of extravasated *TSL-e* is optimal, according to the intended delivery mechanism for TSL-e. Note that this optimal time constant is tied to the 30-min hyperthermia duration. For extended hyperthermia duration longer than 4 h, the optimum release time constant approaches ∼30 min (graph not shown).

#### Validation

A prior in-vivo study in a small animal tumor model measured tissue concentration after administration of different DOX formulations (*Free-DOX*, *Stealth-DOX,* two *TSL-DOX* formulations with varying release rates), both with and without hyperthermia [12]. After adjusting parameters in our model to fit this prior study (TSL release rates, dose), tissue concentration correlates well quantitatively with this prior in-vivo study. Importantly, the prediction for the two *TSL-i-DOX* formulations with different release rates match well. Note that for the fast *TSL-i-DOX* formulation studied above, our model predicts 2.7 times higher tissue concentration than for the slow TSL formulation shown in Figure 12.

#### Toxicities

The administration of all liposomal DOX formulations resulted in significantly reduced peak plasma concentrations and plasma AUC’s of bioavailable drug (Table 2), reduced heart tissue concentration (Table 2), and presumably reduced toxicity, compared to administration of free drug. Cardiac toxicity is of relevance, since it limits lifetime dose of DOX that can be administered. The suggested reduced toxicity indicated by peak cardiac concentration is in accordance with published experimental data [3], [14]. Highest concentration of DOX in cardiac tissue resulted from administration of *Free-DOX* while lowest levels were reached with Stealth liposomes. While the fact that high cardiac concentrations are obtained with free DOX may be contradictory in light of the low tumor concentrations, this can be explained by the very rapid DOX uptake by cardiac cells compared to cancer cells. The ratio between DOX concentration in tumor tissue and heart tissue was best for *TSL-e*, followed by fast-release *TSL-i*, slow-release *TSL-i*, and worst for *Free-DOX* (Figure 11, Table 2). The differences in cardiac tissue uptake are primarily resulting from differences in plasma stability of the various liposomal formulations. Other dose limiting toxicities of DOX, such as myelosuppression, mucositis and PPE, correlate with plasma AUC. Plasma AUC was lowest for Doxil, followed by *TSL-e*, slow *TSL-i*, fast *TSL-i*, optimized Stealth, and highest for *Free-DOX* (Table 2).

#### MDR

Many cancers develop resistance to chemotherapy drugs such as DOX. Literature suggests that MDR is due to presence of membrane proteins (p-glycoprotein and the so-called multidrug resistance-associated protein (MRP)), which facilitate DOX transport out of the tumor cells. We simulated this active efflux mechanism by increasing the transport of DOX from the intracellular tumor compartment to the tumor EES ten times [24]. As expected, the model demonstrated for all DOX formulations a decreased tumor intracellular concentration (Table 2). For Stealth liposomes, optimal release time constant for MDR cells was *τ* = 4 min compared to 45 min for DOX-sensitive cells. In fact, even *Free-DOX* was more effective in MDR cells than the slower *Stealth-DOX* optimized for DOX sensitive cells (*τ* = 45 min) (Figure 8B). Note that the optimal release time constant matches approximately the time constant of cellular uptake (i.e. time it takes for intracellular concentration to saturate), which is in the minute range for MDR cells in our model (uptake time constant data not shown) compared to hours for DOX-sensitive cells [23].

A study that fitted data from several prior in-vitro studies to a cell uptake model found cellular uptake time constants varying between a few minutes to many hours, depending largely on cell type [27]. Combined with our results, this suggests (1) the ideal release time constant may depend on cell type, (2) the release time constant may be tailored to more optimally target a specific cell sub-population, and (3) for optimal delivery to a heterogeneous cell population, a cocktail of carriers with multiple release time constants may be beneficial.

### Conclusion

Slow release Stealth liposomes reduced systemic toxicity, but did not increase antitumor efficacy as measured by maximum intracellular concentration, which is in accordance with clinical observations [43]. Optimized Stealth liposomes resulted in higher tumor intracellular peak concentration, but also in a higher peak plasma concentration and higher plasma AUC, suggesting higher toxicity. The optimal release time constant for Stealth liposomes as well as of *TSL-e* for maximizing intracellular tumor drug concentration depended on cell uptake kinetics, which varies between different types of tumor cells (e.g. MDR status of tumor cells). In addition, optimal release time constant highly depends on drug delivery paradigm (passive accumulation, intra-, or extra-vascular triggered release). Between the compared formulations, *TSL-i* resulted in highest tumor tissue drug concentration by locally keeping plasma concentration of bioavailable DOX high. Note that many of the presented results are directly relevant to other intra- or extra-vascular triggered drug carriers, in addition to TSL. Optimization of liposomal drug delivery systems (in terms of release time constant, plasma half-life, etc.) clearly may improve therapeutic index providing new motivation to the drug delivery community. However, many of these liposomal properties are interdependent, presenting a formidable development challenge. Mathematical models as the one presented here may thus be a valuable tool to aid design and optimization of drug formulations to achieve a better therapeutic index.

## Supporting Information

Equations S1 **Detailed description and equations of mathematical model.**
(DOC)
