# Supplementary material for: Comparison of Conventional Chemotherapy, Stealth Liposomes and Temperature-Sensitive Liposomes in a Mathematical Model
Source: PLoS One. 2012 Oct 17;7(10):e47453. doi: 10.1371/journal.pone.0047453 (PMC3474827; doi:10.1371/journal.pone.0047453)
Supplement: Equations S1 — Detailed description and equations of mathematical model. (DOC) [file pone.0047453.s001.doc]

Detailed description of Mathematical Model:

Temperature sensitive liposomes (TSL-e, TSL-i):

The following system of ordinary differential equations (ODEs) was used to describe the model, all values for the model parameters are shown in Table 1. For simplification the equations for liposomal drug will be presented first, Free-DOX equations will be discussed later as simplified version of the liposomal DOX equations. The equations are described initially for the case of TSL-DOX administration:

The rate of change of liposomal DOX within the systemic plasma compartment is described by:

$\frac{dc_{p\_Lip}}{dt}=-R\cdot c_{p\_Lip}\cdot\frac{V_{p}^{T}}{V_{p}^{B}}-R_{37}\cdot c_{p\_Lip}-k_{e\_LTSL}\cdot c_{p\_Lip}$ (1)

where the first and the second terms account for the decrease of liposomal drug caused by the drug release during heating in the tumor plasma compartment and the release of drug which takes place at 37 °C body temperature in the systemic plasma compartment. The last term accounts for the body clearance of liposomes.

The difference between TSL-i and TSL-e lies in (1) the rate of release, both at body temperature and after heating, (2) differences in plasma stability (which is in part related to release at 37 ºC), (3) the time at which release is triggered (i.e. before, or after considerable tumor tissue accumulation, for TSL-i and TSL-e, respectively), and (4) the location where release occurs (within plasma for TSL-i, and within interstitium for TSL-e).

Stealth-DOX:

Regional heating and localized triggered release, only apply to TSL. Stealth liposomes passively accumulate in the tumor and release their content continuously without trigger. The Stealth formulation releases DOX within the plasma and tumor interstitium (i.e. within the whole body plasma rather than just in the tumor plasma). A constant release rate of liposomal release within plasma as well as interstitium is assumed:

$\frac{dc_{p\_Lip}}{dt}=-k_{rel}c_{p\_lip}-k_{e\_Lip}c_{p\_Lip}$ (2)

where the first term accounts for the decrease of liposomal drug due to released drug and the second term stands for the body clearance of liposomal drug.

Furthermore, in the Stealth-DOX case, in equations 3, 4, 5 and 9, terms regarding liposomal release of DOX, change to *R* = *R_37_* = 1/*τ*, as the release for Stealth-DOX is assumed to be uniform and no heat is applied.

Applicable for both, TSL and Stealth formulations, the rate of change of liposomal DOX within the EES is described by:

$\frac{dc_{e\_Lip}^{T}}{dt}=\frac{1}{v_{e}^{T}}P_{L}S(c_{p\_Lip}-c_{e\_Lip}^{T})-c_{e\_Lip}^{T}R$ (3)

where the first term describes the extravasation of liposomal drug by passive diffusion from the tumor plasma compartment into the tumor EES and the second term accounts for the decrease of liposomal DOX due to the newly released drug.

**Tumor tissue sub-compartments (Unencapsulated DOX concentrations):**

The rate of change of drug concentration in the tumor plasma compartment is described by:

$\frac{dc_{p}^{T}}{dt}=-\frac{1}{v_{p}^{T}}PS_{DOX}(c_{p}^{T}-c_{e}^{T})-F_{pv}^{T}c_{p}^{T}+F_{pv}^{T}c_{p}^{B}+c_{p\_Lip}\cdot R$ (4)

where the first term describes the transvascular transport of DOX by passive diffusion depending on the permeability surface area product *PS*, considering the different volumes of the tumor plasma compartment and the EES, and the second and third term account for the amount of drug which is transported between the plasma tumor compartment and the systemic plasma compartment by blood perfusion (*F_pv_^T^*). The last stands for the drug which is newly released from the liposomes within the plasma tumor compartment.

Equation 5 describes the rate of change of DOX within the tumor EES:

$\frac{dc_{e}^{T}}{dt}=\frac{1}{v_{e}^{T}}PS_{DOX}(c_{p}^{T}-c_{e}^{T})-k_{3ci}(k_{1ci}c_{e}^{T}+\frac{k_{2ci}c_{e}^{T}}{K_{ici}+c_{e}^{T}}-k_{5ci}c_{i}^{T})+c_{e\_Lip}^{T}\cdot R$ (5)

where the first term accounts for the transvascular transport of DOX between the tumor plasma compartment and the tumor EES, and the second term decreases the DOX concentration in the EES for the amount of drug which is taken up by cells (see Equation 6). The last term accounts for the amount of DOX which is newly released by the liposomes within the tumor EES.

$\frac{dc_{i}^{T}}{dt}=k_{3ci}(k_{1ci}c_{e}^{T}+\frac{k_{2ci}c_{e}^{T}}{K_{ici}+c_{e}^{T}}-k_{5ci}c_{i}^{T})$ (6)

Equation 6 describes the rate of change of DOX within the intracellular tumor compartment. The intracellular uptake model of [1] was used. There are two different concurrent intracellular uptake mechanisms of DOX: passive diffusion across the cell membrane, and an active transport mechanism, which is most likely endocytosis. Note that the intracellular uptake model likely varies for different cell types. In this model data for small human lung cancer cells [2] were used. The constant *k_5ci_* was added to simulate additional the case of MDR resistance of cancer cells to simulate an increased drug efflux out of the cells. *k_5ci_*=1 in the case of DOX sensitive cells and *k_5ci_*=10 [3] in the case of MDR cells.

The total tumor tissue drug concentration is calculated by adding the tumor EES (unencapsulated and liposomal) and the tumor intracellular DOX concentration, considering the volume fractions of each compartment:

*ct_total^T^*=*c_i_^T^v_i_^T^* + *c_e_^T^v_e_^T^* + *ce_Lip^T^v_e_^T^* (7)

The bioavailable tumor tissue drug concentration is calculated by adding the tumor EES and the tumor intracellular unencapsulated DOX concentration, considering the volume fractions of each compartment:

*ct_bioavailable^T^*=*c_i_^T^v_i_^T^* *+ c_e_^T^v_e_^T^* (8)

**Body and heart tissue compartments:**

The systemic plasma compartment concentration considers inflow and outflow from tumor, as well as clearance and uptake by body tissue. The rate of change of drug in the systemic plasma compartment is described by:

$\frac{dc_{p}^{B}}{dt}=\frac{V_{p}^{T}}{V_{p}^{B}}F_{pv}^{T}(c_{p}^{T}-c_{p}^{B})-k_{e}c_{p}^{B}-k_{p}c_{p}^{B}+k_{t}c_{t}^{B}+c_{p\_Lip}\cdot R_{37}$ (9)

where the first term on the right describes the transport of drug between the systemic plasma compartment and the tumor plasma compartment due to the perfusion of blood (*F_pv_^T^*), considering the different compartment volumes, the second term describes clearance, third and fourth terms account for uptake by lump body tissue compartment. The last term accounts for the amount of drug which is newly released by the liposomes within the systemic plasma compartment at 37 °C. Note that the lump tissue compartment includes the heart, and therefore uptake by heart tissue is not included in equation 9. Heart tissue is however separately modeled below, to serve as measure of cardiac toxicity.

The rate of change of DOX in the lump body tissue compartment is then described by:

$\frac{dc_{t}^{B}}{dt}=k_{p}c_{p}^{B}-k_{t}c_{t}^{B}$ (10)

Equation 11 describes the rate of change in the heart tissue compartment:

$\frac{dc_{t}^{H}}{dt}=k_{ph}c_{p}^{B}-k_{hp}c_{t}^{H}$ (11)

where the two terms account for the concentration of drug which is transported between the systemic plasma compartment and the cardiac tissue compartment.

The initial concentrations of drug in the case of TSL-DOX, as well as for Stealth-DOX administration are: *c_p_^B^* = *c_t_^B^* = *c_t_^H^* = *c_p_^T^* = *c_e_^T^* = *c_i_^T^* = *ce_Lip^T^* = 0; *cp_Lip(t=0) = D*BW/V_p_^B^*.

In the case of Free-DOX administration there is no liposomal drug, consequently Equation 1 and Equation 2 are not applicable, and the terms for liposomal release in equations 4, 5 and 9 are eliminated. For Free-DOX administration the initial concentrations of DOX are: *c_p_^T^*  = *c_e_^T^* = *c_i_^T^* = *c_t_^B^* = *c_t_^H^* = 0; *c_p_^B^(t=0)* = *D*BW/VD*.

Table 1. Complete list of model parameters

| **Symbol** | **Description** | **Value** | **Source** |
| --- | --- | --- | --- |
| *BW* | Body weight for mice | 20 g | assumed |
| *VD* | Volume of distribution | 19e-6 m^3^ | Calculated with data from [4] |
| *D* | Total dose of encapsulated DOX injected | 0.18 mg | 9 mg/kg * bodyweight (assumed ) |
| *Hct* | Hematocrit | 0.45 | [5] |
| *Hct_tumor_* | Hematocrit for tumor microvasculature | 0.19 | [6] |
| *k_p_* | Transfer constant from systemic plasma to lump tissue | 9.4e-3 s^-1^ | calculated with data from [4] |
| *k_e_* | Transfer constant for clearance | 2.1e-3 s^-1^ | calculated with data from [4] |
| *k_t_* | Transfer constant from tissue to systemic plasma | 7.052e-5 s^-1^ | calculated with data from [4] |
| *k_ph_^a^* | Transfer constant from systemic plasma to heart tissue | 8.3e-3 s^-1^ | calculated with data from [4] |
| *k_hp_^a^* | Transfer constant from heart tissue to systemic plasma | 1.1481e-4 s^-1^ | calculated with data from [4] |
| *ke_TSL* | Rate constant of TSL clearance | 2.228e-4 s^-1^ | fit to data from [7] |
| *k_1ci_* | Parameter for intracellular uptake | 2.257 | [1] |
| *k_2ci_* | Parameter for intracellular uptake | 0.0452 kg/m^3^ | [1] |
| *k_3ci_* | Parameter for intracellular uptake | 2.806e-4 s^-1^ | [1] |
| *k_5ci_* | Parameter for active efflux of DOX from intracellular compartment without MDR  in case of MDR | 1  10 | [3] |
| *K_ici_* | Parameter for intracellular uptake | 5.29e-4 kg/m^3^ | [1] |
| *PS_DOX_* | Permeability surface area product for DOX | 4.9e-3 s^-1^ | [8] |
| *V_B_^B^* | Total blood volume in body | 0.98 mL | calculated with: blood = 4.9% of body weight for mice [9] |
| *V_p_^B^* | Volume of systemic plasma | 0.54 mL | *V_B_^B^*(1-*Hct*) |
| *v_v_^T^* | Volume fraction of tumor vascular space | 0.092 | [10] |
| *v_p_^T^* | Volume fraction of tumor plasma space | 0.0745 | *v_v_^T^*(1-*Hct_tumor_*) |
| *v_e_^T^* | Volume fraction of tumor EES | 0.454 | [11] |
| *v_i_^T^* | Volume fraction of tumor intracellular space | 0.454 | (1-*v_v_^T^*-*v_e_^T^*) |
| *R* | Release rate of DOX from TSL-i during heat,  for fast TSL-i if *F_pv_^T^*>*R*  for slow TSL-1 if *F_pv_^T^*>*R*  if *F_pv_^T^*<=*R* | 0.3 s^-1^  0.013 s^-1^  F_pv_^T^ | calculated |
| *R_37_* | Release rate of DOX from TSL-i at 37 °C | variable [s^-1^] | calculated |
| *ρ_Tissue_* | Density for liver tissue | 1060 kg/m^3^ |  |
| *w_0_* | Blood perfusion | 108 ml/100 ml/min | [12] |
| *BV* | Blood Volume in liver parenchyma | 0.06678 | (6.3 g / 100 ml * *ρ_Tissue_*) [13] |
| *F_pv_^T^* | Plasma Flow in tumor plasma space; note: *F*_pv_=plasma flow/plasma volume | variable [s^-1^] | *w_0_*/*BV* |
| *P_L_S* | Permeability surface area product for liposomes | 6.8e-5 s^-1^ | [14, 15] |
| *V^T^* | Volume of tumor with tumor diameter=2e-3 m | 4.189e-3 mL |  |
| *V_p_^T^* | Volume of tumor plasma | 3.12e-4 mL | *V^T^***v_p_^T^* |
| *τ* | Time constant for DOX release from Doxil (equal for plasma and EES) | 454.4 h | [16, 17] |
| *k_rel_* | release rate of DOX from Stealth liposomes | 0.002 h^-1^ | *k_rel_*=1/*τ* |
| *ke_Lip* | clearance Stealth liposomes | 0.0339 h^-1^ | [16] |

^a^To calculate k_ph_ and k_hp_, a two compartment model, representing the concentrations of DOX in the body plasma and the heart tissue compartment, was assumed. To estimate the accurate values for the rate constants the simplex method was used.

**References**

[1] A.W. El-Kareh, T.W. Secomb, Two-mechanism peak concentration model for cellular pharmacodynamics of Doxorubicin. Neoplasia 7(7) (2005) 705-713.

[2] D.J. Kerr, A.M. Kerr, R.I. Freshney, S.B. Kaye, Comparative intracellular uptake of adriamycin and 4'-deoxydoxorubicin by non-small cell lung tumor cells in culture and its relationship to cell survival. Biochem Pharmacol 35(16) (1986) 2817-2823.

[3] J.P. Sinek, S. Sanga, X. Zheng, H.B. Frieboes, M. Ferrari, V. Cristini, Predicting drug pharmacokinetics and effect in vascularized tumors using computer simulation. J Math Biol (2008).

[4] W.J. van der Vijgh, P.A. Maessen, H.M. Pinedo, Comparative metabolism and pharmacokinetics of doxorubicin and 4'-epidoxorubicin in plasma, heart and tumor of tumor-bearing mice. Cancer Chemother Pharmacol 26(1) (1990) 9-12.

[5] P.S. Tofts, G. Brix, D.L. Buckley, J.L. Evelhoch, E. Henderson, M.V. Knopp, H.B. Larsson, T.Y. Lee, N.A. Mayr, G.J. Parker, R.E. Port, J. Taylor, R.M. Weisskoff, Estimating kinetic parameters from dynamic contrast-enhanced T(1)-weighted MRI of a diffusable tracer: standardized quantities and symbols. J Magn Reson Imaging 10(3) (1999) 223-232.

[6] D.M. Brizel, B. Klitzman, J.M. Cook, J. Edwards, G. Rosner, M.W. Dewhirst, A comparison of tumor and normal tissue microvascular hematocrits and red cell fluxes in a rat window chamber model. Int J Radiat Oncol Biol Phys 25(2) (1993) 269-276.

[7] R.T. Poon, N. Borys, Lyso-thermosensitive liposomal doxorubicin: a novel approach to enhance efficacy of thermal ablation of liver cancer. Expert Opin Pharmacother 10(2) (2009) 333-343.

[8] T.L. Jackson, Intracellular accumulation and mechanism of action of doxorubicin in a spatio-temporal tumor model. J Theor Biol 220(2) (2003) 201-213.

[9] R.P. Brown, M.D. Delp, S.L. Lindstedt, L.R. Rhomberg, R.P. Beliles, Physiological parameter values for physiologically based pharmacokinetic models. Toxicol Ind Health 13(4) (1997) 407-484.

[10] F. Yuan, M. Leunig, D.A. Berk, R.K. Jain, Microvascular permeability of albumin, vascular surface area, and vascular volume measured in human adenocarcinoma LS174T using dorsal chamber in SCID mice. Microvasc Res 45(3) (1993) 269-289.

[11] R.K. Jain, Transport of molecules in the tumor interstitium: a review. Cancer Res 47(12) (1987) 3039-3051.

[12] D.J. Schutt, D. Haemmerich, Effects of variation in perfusion rates and of perfusion models in computational models of radio frequency tumor ablation. Med Phys 35(8) (2008) 3462-3470.

[13] H. Kojima, N. Tanigawa, A. Komemushi, S. Kariya, S. Sawada, Computed tomography perfusion of the liver: assessment of pure portal blood flow studied with CT perfusion during superior mesenteric arterial portography. Acta Radiol 45(7) (2004) 709-715.

[14] L.T. Baxter, R.K. Jain, Transport of fluid and macromolecules in tumors. I. Role of interstitial pressure and convection. Microvasc Res 37(1) (1989) 77-104.

[15] N.Z. Wu, B. Klitzman, G. Rosner, D. Needham, M.W. Dewhirst, Measurement of material extravasation in microvascular networks using fluorescence video-microscopy. Microvasc Res 46(2) (1993) 231-253.

[16] T.M. Allen, D.R. Mumbengegwi, G.J. Charrois, Anti-CD19-targeted liposomal doxorubicin improves the therapeutic efficacy in murine B-cell lymphoma and ameliorates the toxicity of liposomes with varying drug release rates. Clin Cancer Res 11(9) (2005) 3567-3573.

[17] K.M. Laginha, S. Verwoert, G.J. Charrois, T.M. Allen, Determination of doxorubicin levels in whole tumor and tumor nuclei in murine breast cancer tumors. Clin Cancer Res 11(19 Pt 1) (2005) 6944-6949.
